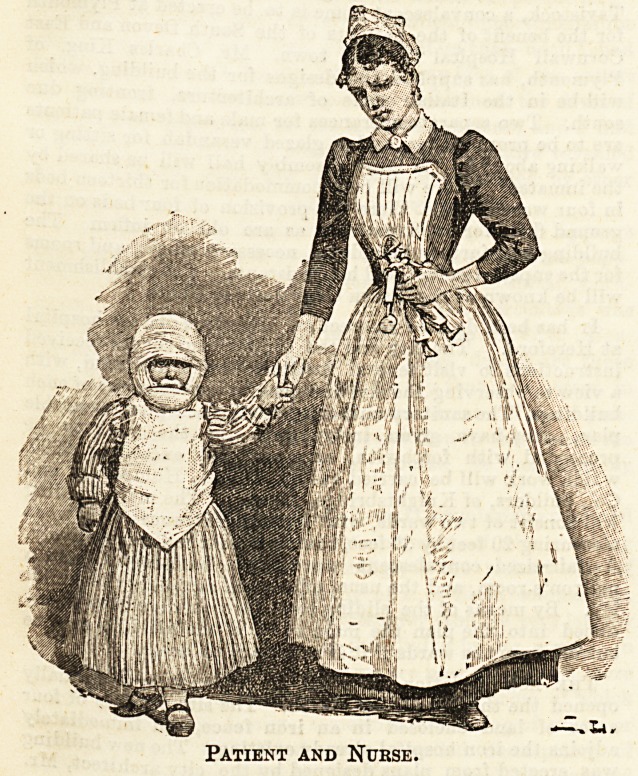# New Hospital for Children at Leipzig

**Published:** 1893-04-08

**Authors:** 


### The Institutional Workshop.

### HOSPITAL CONSTRUCTION.

### NEW HOSPITAL FOR CHILDREN AT LEIPZIG.

The new Children's Hospital at Leipzig, situated in the east-end of the town, is certainly an ornament to the city. It consists of six separate buildings of bright red brick, and all

Patient and Nurse.

are surrounded by well-arranged gardens with luxuriant grass lawns interspersed. The whole place is an evidence of generosity on the part of present and also of former citizens, and it justifies the universal pride felt in its successful completion.

There is only one house which can be entered from the street, and this, known as the west wing, is merely divided by a garden from the thoroughfare. The receiving room is situated in the centre of this block, and passing through it the out-patient department is soon reached, and also the governor's room.

To the right of the receiving-room lies the principal building, which is decorated by coloured cross-beams and verandahs. It contains a large hall, and also one of the wards. The block for infectious diseases is at the far side of the square, exactly opposite the principal building. It consists of three small houses for diphtheria, scarlet fever, and measles. The housekeeping department is opposite the west wing, and it, like every other part of the building, has a pleasant inviting appearance, both without and within.

It is more than a year since the new establishment opened its doors to receive patients, and much good work has already been done within the walls. The ventilation is well managed, and the wards are flooded with air and sunshine, those powerful antagonists of misery and disease.

A spacious day-room is situated between two of the wards and convalescents and others for whom the removal is permitted, adjourn to it for play and for the mid-day meal.

On Sundays and Wednesdays the parents of the children are allowed to visit them, and to bring toys if they like, but the introduction of any kind of food is prohibited. The doctors attend from two to four daily in the out-patient department, which is a busy scene at that time, over a hundred persons frequently assembling in the waiting-hall with children of all ages.

Only three wards are absolutely "free," and therefore the parents of little patients nursed in the hospital usually give a donation.

## Figures and Tables

**Figure f1:**